# Targeting senescent microglia in progressive multiple sclerosis: a geroscience-informed approach

**DOI:** 10.3389/fimmu.2025.1681724

**Published:** 2025-10-07

**Authors:** Jeffrey Atkinson, Amy Dokiburra, Hayley Groover, Jonathan P. Godbout, Benjamin M. Segal, Yinan Zhang

**Affiliations:** ^1^ Department of Neurology, The Ohio State University College of Medicine, Wexner Medical Center, Columbus, OH, United States; ^2^ The Neuroscience Research Institute, The Ohio State University Wexner Medical Centers, Columbus, OH, United States; ^3^ Neuroscience Graduate Program, The Ohio State University, Columbus, OH, United States; ^4^ Department of Neuroscience, The Ohio State University Wexner Medical Center, Columbus, OH, United States; ^5^ Chronic Brain Injury Program, The Ohio State University, Columbus, OH, United States

**Keywords:** multiple sclerosis, senolytic, geroscience, aging, experimental autoimmue encephalomyelitis

## Abstract

Multiple sclerosis (MS) is a neuroinflammatory and neurodegenerative disorder of the central nervous system (CNS). Age is the strongest predictor of disease phenotype, with the majority of older adults transitioning to a progressive form marked by irreversible neurological decline. This clinical progression is associated with smoldering, CNS-compartmentalized inflammation and neurodegeneration, for which there are currently no effective disease-modifying therapies. Cellular senescence, characterized by the secretion of pro-inflammatory mediators collectively known as the senescence-associated secretory phenotype (SASP), increases with age and contributes to tissue injury. In MS, neuroinflammation can further promote cellular senescence, creating a self-reinforcing cycle of damage. Senescent microglia have been identified within MS lesions, where their SASP may impair remyelination and exacerbate neurodegeneration. Senolytic agents selectively target and eliminate senescent cells by disrupting anti-apoptotic pathways. In experimental autoimmune encephalomyelitis (EAE), a widely used model of MS, senolytic treatment reduces senescent microglia burden and attenuates disease severity in an age- and drug-dependent manner. Specifically, here we show that middle-aged mice (40–44 weeks) with EAE exhibit improved clinical outcomes and survival following treatment with either dasatinib plus quercetin (D+Q) or navitoclax. Early-phase clinical trials of senolytics in age-related diseases have demonstrated functional benefits, including improved gait speed in idiopathic pulmonary fibrosis and CNS penetrance in Alzheimer’s disease. Translating senolytic therapy to MS will require careful selection of CNS-penetrant and well-tolerated agents, identification of appropriate patient populations, and deployment of responsive biomarkers. Senolytic therapy represents a promising geroscience-based strategy to meet the urgent therapeutic need in progressive MS.

## Introduction

Multiple sclerosis (MS) is a relapsing or chronic progressive multifocal inflammatory and neurodegenerative disorder of the central nervous system (CNS), which afflicts approximately one million people in the United States (1). Age is the strongest predictor of MS clinical course (2–5). The disease typically presents during young adulthood as relapsing-remitting MS (RRMS), characterized by self-limited episodes separated by clinically quiescent periods. These relapses are associated with acute inflammatory demyelinating white matter lesions, driven by focal blood-brain-barrier breakdown and infiltration of peripheral leukocytes into the perivascular space and parenchyma. As people with RRMS age, up to 90% transition to a progressive form of the disease, defined by the gradual accrual of irreversible neurological disability (6, 7). Progressive MS is pathologically distinguished by smoldering, CNS-compartmentalized inflammation behind an intact blood–brain barrier, slowly expanding lesions, widespread microglial activation, and structured meningeal inflammation (8). Currently, more than 20 FDA-approved disease-modifying therapies (DMTs) are available for RRMS, primarily targeting the peripheral immune system to prevent relapses and new lesion formation (9). However, these therapies are largely ineffective in halting or reversing disability progression in progressive MS (10). This highlights an urgent unmet need for novel therapeutic strategies that specifically address the pathophysiology of progressive MS. One promising approach is the application of geroscience-based therapies that address age-related mechanisms of CNS dysfunction. We summarize emerging evidence of senescent cells, in particular senescent microglia, contributing to neurodegeneration in progressive MS and the effects of their clearance in animal models of MS. We describe translational considerations of using senolytics in people with MS based on lessons learned and challenges observed in early phase senolytic trials in other age-related conditions.

## Senescent microglia as a driver of MS progression

Cellular senescence is a hallmark of aging, characterized by the permanent exit of cells from the cell cycle and accompanied by distinct morphological and functional changes in response to cellular damage and stress (11). Senescent cells remain metabolically active and secrete a panel of pro-inflammatory mediators collectively known as the senescence-associated secretory phenotype (SASP), which drives chronic low-grade systemic inflammation (12, 13), and contributes to impaired tissue regeneration and the onset of age-related diseases (14). Premature accumulation of senescent cells is a feature of neurodegenerative diseases such as Alzheimer’s and Parkinson’s disease, with emerging evidence suggesting a similar phenomenon in progressive MS (15–18).

Buildup of senescent cells in the CNS creates oxidative stress that hinders the remyelinating capacity of oligodendrocyte progenitor cells and promotes neurodegeneration (16, 19). In progressive MS, senescent cells exhibiting the SASP accumulate in actively demyelinating gray and white matter lesions and is associated with faster disability progression and higher mortality (20). SASP components can mediate axonal damage and impede myelin repair (19). Senescent cells express high levels of the tumor suppressor gene *p16^INK4a^* (21, 22), and postmortem analysis of MS lesions has revealed a greater density of *p16^INK4a^*-positive cells compared to normal white matter (23). In *p16^INK4a^*-luciferase reporter mice, bioluminescence signal from aged brains correlated with elevated *p16^INK4a^* mRNA and protein expression in white matter regions (24). Single-cell RNA sequencing identified microglia as the primary cell type expressing *p16^INK4a^* in the aged brains. Isolated microglia from aged brains corroborated increased *p16^INK4a^* mRNA and protein expression. Moreover, these senescent microglia showed reduced proliferative potential, as evidenced by diminished 5-ethynyl-2’-deoxyuridine (EdU) incorporation compared to *p16^INK4a^*-negative microglia. In experimental autoimmune encephalomyelitis (EAE), a widely used animal model of MS, *p16^INK4a^*-positive microglia accumulate in the spinal cord at the peak of clinical disease severity. Notably, *p16^INK4a^*-deficient mice exhibited reduced CNS infiltration by T cells, less demyelination and attenuated clinical disability (24). Collectively these findings indicate that microglia are the predominant senescent cell in CNS white matter during aging and EAE.

Recent studies have investigated the relationships between cellular senescence, microglial proliferation and remyelination in the EAE model. Compared to young adult mice, aged mice with EAE exhibited a more severe clinical course, increased neurodegeneration, and impaired remyelination (25). Spatial gene expression profiling of spinal cord lesions revealed an expansion of macrophages and microglia, reduced oligodendrocyte density, and upregulation of senescence-associated gene signatures—changes that were especially pronounced in aged mice (26). The accumulation of senescent cells within lesions was accompanied by a marked reduction in oligodendrocyte precursor cell (OPC) differentiation and mature oligodendrocyte formation. Senescent cells could, potentially, modulate oligodendroglial cells through the production of soluble factors. Proteomic analysis of CNS homogenates has demonstrated that CCL11/Eotaxin-1, a chemokine involved in myeloid cell recruitment and a component of the SASP, is elevated in the spinal cords of aged mice with EAE. Plasma CCL11 levels are elevated in progressive MS and correlate with disease severity (27). It readily crosses the blood–brain barrier and has been shown to suppress myelin basic protein (MBP) expression in maturing oligodendrocytes (26, 28).

The hyperproliferation of certain microglial subsets may lead to replication stress and the induction of senescence programs (29). When coupled with age-related impairments in the clearance of senescent cells, this results in the accumulation of dysfunctional, senescent microglia within the CNS. Collectively, these findings support a model in which microglial senescence contributes to remyelination failure and neurodegeneration, thereby accelerating disease progression in progressive MS.

## Senolytics remove senescent microglia

Current treatment of progressive MS in older adults focuses on palliative symptom management rather than addressing the underlying mechanisms such as age-related immune dysregulation. The association between cellular senescence and age-related diseases highlights the critical role of aging mechanisms in clinical outcomes of age-related diseases (30). Although chronological age is the greatest risk factor for MS disease progression (2–5), there is variation in the age at which patients with RRMS convert to SPMS, which may be explained by interindividual variations in biological aging mechanisms (30) (31).

Senescent cells can be selectively eliminated by senolytic drugs, which have been shown to delay age-related decline in animal models and hold promise for improving functional outcomes in human clinical trials (32). The discovery of senolytics was guided by the observation that senescent cells upregulate anti-apoptotic pathways to resist cell death. Proteomic and transcriptomic analyses of senescent cells revealed upregulation of antiapoptotic pathways, and disabling these antiapoptotic pathways selectively depletes these cells while sparing non-senescent cells (32). The first senolytics identified were dasatinib (D), a tyrosine kinase inhibitor, and quercetin (Q), a naturally occurring flavonoid. In combination (D+Q), these agents induce apoptosis in senescent cells across multiple tissues (33). Dasatinib, a FDA-approved cancer treatment, disrupts pro-survival signaling in senescent cells (34, 35), while quercetin impairs DNA damage repair and inhibits the PI3K/AKT pathway, promoting senescent cell death (36, 37). Together, D+Q act synergistically to eliminate a broader range of senescent cells than either agent alone (38), leading to improved lifespan in preclinical studies in mice (39). Notably, clearance of peripheral senescent cells has been linked to improved outcomes in CNS disorders (40). The removal of senescent cells by senolytics underscores their therapeutic potential.

Targeting senescent microglia represents a promising strategy for progressive MS, a disease stage characterized by limited blood-brain barrier disruption and minimal peripheral immune infiltration. A recent study demonstrated accumulation of p21^+^ senescent-like myeloid cells—including monocyte-derived macrophages and microglia—in the spinal cord and meninges during EAE (41). Treatment of EAE mice with D+Q reduced the frequency of these senescent-like myeloid cells, but did not impact clinical disease severity. A possible explanation for the lack of efficacy may be due to the exclusive use of young female mice in this study, wherein it was previously shown that D+Q displayed sexual dimorphic treatment effects in young adult mice (42). Alternatively, according to the threshold theory of senescence, senescent cell burden in young animals may remain below the threshold necessary to cause clinical pathology (32).

**Figure 1:**
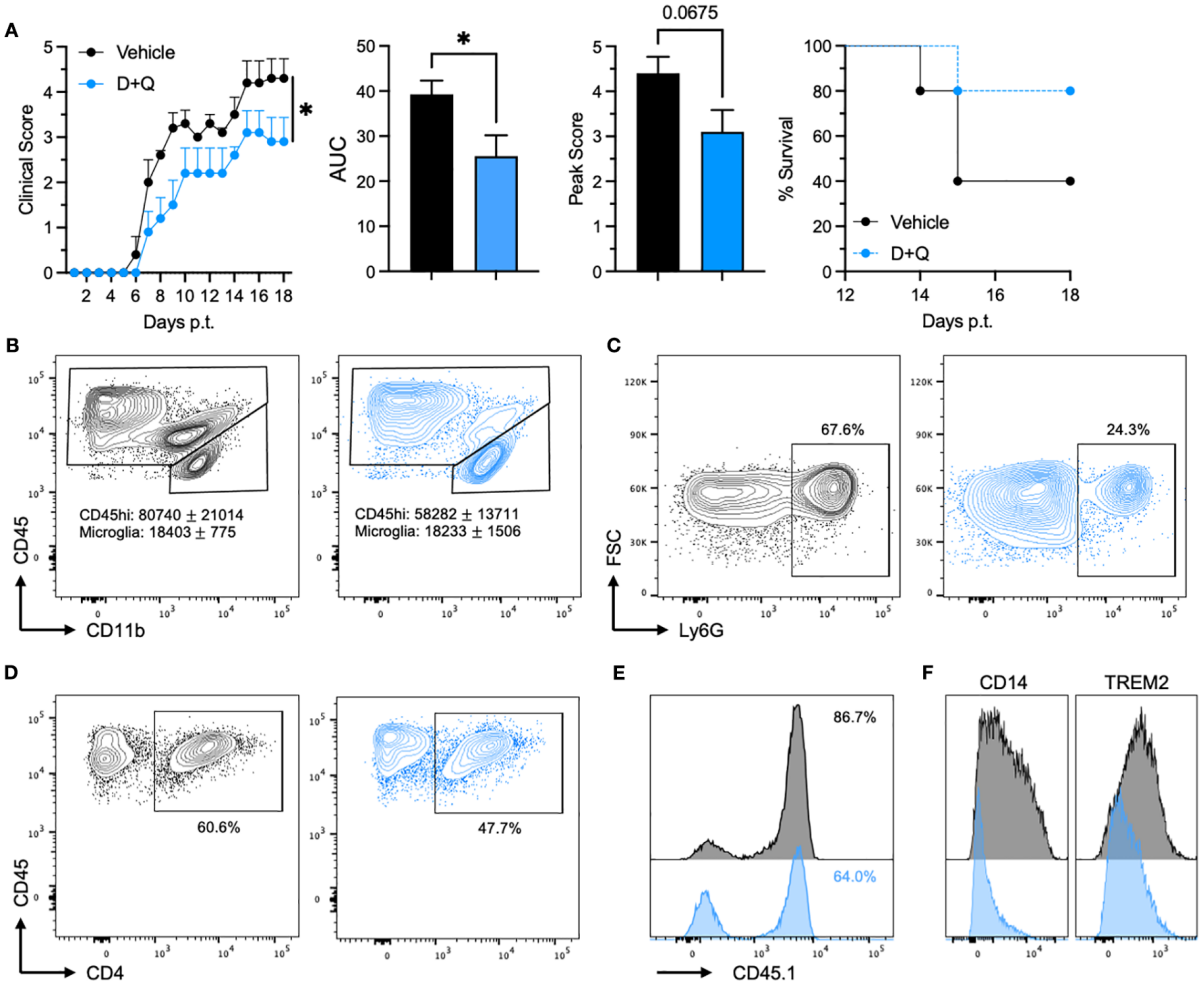
Dasatinib and quercetin (D+Q) treatment ameliorates EAE and reduces senescent microglial phenotypes in middle-aged mice. **(A)** Clinical course of EAE in middle-aged mice treated with vehicle (black, *n* = 5) or D+Q (blue, *n* = 5), starting at symptom onset. Dasatinib (5 mg/kg; HY-10181, MedChemExpress) and Quercetin (50 mg/kg; HY-18085, MedChemExpress) suspended in a 10% DMSO/30% PEG300/10% Tween80/50% water solution, or vehicle solution alone, was administered i.v. into the tail vein. Left: daily mean clinical scores; Middle left: area under the clinical score curve (AUC); Middle right: mean peak clinical scores; Right: survival over time. **(B–E)** Flow cytometric analysis of immune cells harvested from the spinal cords of vehicle-treated (black) and D+Q-treated (blue) EAE mice on day 18 post-transfer. **(B–D)** Representative contour plots of infiltrating peripheral immune cells (CD45^hi^) and microglia (CD11b^+^ CD45^int^), gated on all viable CD45+ cells, **(B)** Ly6G+ neutrophils, gated on CD45hi CD11b+ infiltrating peripheral myeloid cells, **(C)**, and CD4+ T cells, gated on CD45+ CD11b- cells **(D)**. **(E)** Representative histograms showing CD45.1 expression gated on total CD4+ T cells. The percentage of CD45.1+ donor encephalitogenic T cells is shown. **(F)** Representative histograms showing expression of CD14 (left) and TREM2 (right) on microglia, gated as in **(B)** All experiments were performed with female C57BL/6 mice purchased from Charles River. Statistical significance was determined using unpaired 2-tailed Student’s t test. **(A)** Curves in the left panel were compared using a 2-way ANOVA. Error bars indicate mean ± SEM. *p < 0.05, or as indicated.

In our own study, using Th17-mediated adoptive transfer EAE, we found that initiating daily D+Q treatment at symptom onset (approximately day 6 following T cell transfer) significantly ameliorated disease in middle-aged (40–44 weeks old) mice compared to vehicle-treated controls (**Figure 1A**). Treated mice exhibited lower peak clinical scores, reduced cumulative disease severity (AUC), and doubled survival rates. D+Q treatment also led to diminished infiltration of peripheral CD45^hi^ immune cells into the inflamed spinal cord, including neutrophils and encephalitogenic CD45.1^+^ CD4^+^ T cells (**Figures 1B–E**). Prior work from our group strongly implicates microglia as a key driver of age-dependent progressive-like disease (25). Notably, D+Q treatment reduced microglial expression of CD14 and TREM2 (**Figure 1F**), markers previously associated with a senescent microglial phenotype (43, 44). These data show D+Q may selectively improve clinical outcomes in middle aged rather than young EAE mice.

A separate study demonstrated the efficacy of the senolytic navitoclax in attenuating EAE severity. Single-cell RNA sequencing at peak disease revealed a microglial population with transcriptional signatures of inflammation, neurodegeneration, and senescence (45). Among these, anti-apoptotic BCL2-family genes, including of *BCL2L1*, were differentially expressed in EAE microglia compared to control counterparts, but not in other CNS cell types. Notably, *BCL2L1* ^+^ microglia exhibited higher expression of proinflammatory genes compared to *BCL2L1* ^−^ microglia. Similar patterns were observed in human MS tissue, where microglia within chronic active lesions showed features of senescence and enrichment for *BCL2L1* transcripts.

Treatment of EAE mice with navitoclax, a BCL2 family inhibitor, selectively depleted senescent microglia and macrophages in the spinal cord without altering the proportions of other cell types (45). Treated animals exhibited sustained improvements in motor function beginning two days post-treatment, along with preserved visual acuity. Histological analysis of optic nerves revealed reduced inflammation and gliosis, accompanied by increased survival of retinal ganglion cells.

**Figure 2:**
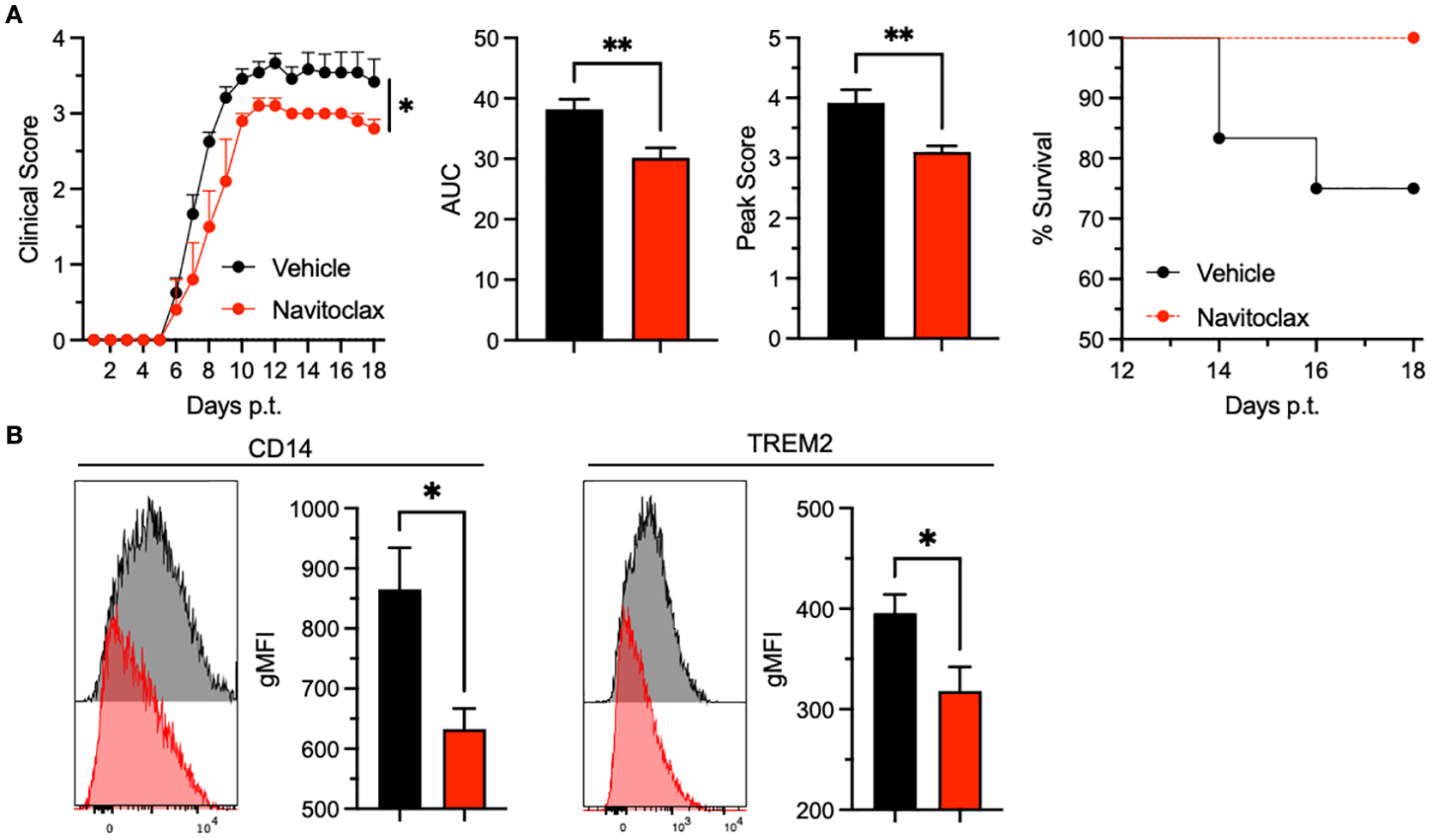
Navitoclax treatment ameliorates EAE and reduces senescent microglial phenotypes in middle-aged mice. **(A)** Clinical course of EAE in middle-aged mice treated with vehicle (black, *n* = 12) or navitoclax (red, *n* = 5), starting at symptom onset. Navitoclax (5 mg/kg; HY-10087, MedChemExpress) suspended in a 10% DMSO/30% PEG300/10% Tween80/50%water solution, or vehicle solution alone, was administered i.v. into the tail vein. **(A)** Clinical score curve for middle-aged mice treated with either vehicle (black, n=12) or navitoclax (red, n=5) beginning at the time of clinical disease onset. Left: daily mean clinical scores; Middle left: area under the clinical score curve (AUC); Middle right: mean peak clinical scores; Right: survival over time. **(B)** Histograms and corresponding geometric mean fluorescent intensity (gMFI) showing the expression of CD14 (left) and TREM2 (right), gated on CD45^int^ CD11b^+^ microglia. Data are representative of 2 individual experiments. All experiments were performed with female C57BL/6 mice purchased from Charles River. Statistical significance was determined using unpaired 2-tailed Student’s t test. Curves in the left panel of A were compared using a mixed effects model. Error bars indicate mean ± SEM. *p < 0.05, **p < 0.01.

Our group has extended these findings to middle-aged mice, where navitoclax treatment similarly reduced overall disease severity, peak clinical scores, and mortality (**Figure 2A**). These clinical improvements were associated with decreased expression of senescence-associated markers in spinal cord microglia (**Figure 2B**), mirroring the effects observed with D+Q treatment. Together, these data highlight the therapeutic potential of senolytic agents in EAE—particularly in the aged CNS—through selective elimination of senescent microglia.

## Towards clinical trials of senolytics in MS

Currently, there are no effective treatments that reliably halt, yet alone slow, disability accumulation in progressive MS. Traditional approaches to understanding MS pathophysiology and therapy have largely overlooked the influence of aging, which may critically shape disease mechanisms and treatment responses. Although several recent publications have proposed targeting cellular senescence in MS using senolytic agents (15–18), no clinical trials of senolytics for MS have been published.

Clinical trials of senolytics have been conducted in other age-related diseases. In the first-in-human senolytic trial, 14 patients with idiopathic pulmonary fibrosis were treated and showed improved gait speed and increased chair-stand repetitions, indicating enhanced physical function (46). A phase 1 study of D+Q in people with Alzheimer’s disease demonstrated that dasatinib crosses the blood–brain barrier. Although therapeutic efficacy was not demonstrated, the study showed that the combination therapy is safe and well-tolerated, supporting advancement to placebo-controlled efficacy trials (47), which are currently underway (NCT04685590; NCT04785300). As a repurposed drug, D+Q has demonstrated acceptable safety profiles and enables faster track to clinical trials. The combination of D+Q targets more anti-apoptotic pathways than either drug alone, resulting in removal of more senescent cells from different tissues (38). Removal of senescent cells in the periphery of individuals with CNS disorders has been linked to clinical improvement (40). A phase 2 study of D+Q in postmenopausal women demonstrated that individuals with higher baseline senescent cell burden—measured by *p16^INK4a^* expression in T cells—exhibited a greater therapeutic response to D+Q, resulting in increased bone mineral density (48). Although other senolytics have been tested in preclinical models, D+Q remain the most commonly used senolytics in clinical trials due to acceptable safety and tolerability (49).

Because senescent cells are non-proliferative and require weeks to reaccumulate and reestablish the SASP, they do not require continuous inhibition of pro-survival pathways for sustained therapeutic benefit (38, 50, 51). A short course of D+Q treatment in people with diabetic kidney disease reduced senescent cell burden and maintained the effect for at least 11 days (52), showing senolytics can be dosed every 2 weeks while maintaining efficacy. The short half-life (<11 hours) of D+Q coupled with the intermittent dosing in a “hit and run” approach minimizes drug exposure and lowers the risk of off-target and side effects (46).

There are challenges in the design of clinical trials of senolytics for MS. Most notably, there are no standards for identifying and monitoring of senescent cells, and standardized biomarkers of senescent cell accumulation and removal are still in development. Some biomarkers may be helpful. For example, the treatment effects of D+Q can be measured using the senescence biomarker *p16^INK4a^*, a tumor suppressor marker that is a key trigger of cellular senescence (53, 54). Senescent cells express high levels of the tumor suppressor gene *p16^INK4a^* (21, 22), and higher levels have corresponded to increased treatment effect with senolytics (48). Nevertheless, *p16^INK4a^* expression is not exclusive to senescent cells as it is upregulated in other terminally differentiated immune cells (55, 56), limiting its usefulness as a selective marker of senescent cells. SASP factor panels and composite biomarker scores are also likely to be helpful in defining response to senolytic treatment. In progressive MS, it is possible that senescence of other immune cell types besides microglia contribute to disease progression. Aging in MS is associated with premature immune exhaustion, marked by an accumulation of effector memory CD4^+^ and CD8^+^ T cells and disrupted regulation of immunoregulatory and costimulatory pathways. Compared to age- and sex-matched controls, individuals with MS exhibit a higher frequency of effector memory T cells and a reduced frequency of naïve T cells in both CD4^+^ and CD8^+^ circulating compartments (57). Furthermore, unlike individuals without MS, those with MS exhibit a reduced frequency of CTLA-4^+^ memory T cells, which play a key role in regulating immune activation and have been implicated as contributors to progressive MS (57). Senescence has also been observed in neural progenitor cells (NPCs) derived from induced pluripotent stem cells of patients with primary progressive MS (19). These NPCs expressed markers of cellular senescence and failed to support oligodendrocyte progenitor cell maturation, in contrast to NPCs derived from age-matched control lines. Treatment with rapamycin restored the ability of senescent NPCs to support oligodendrocyte maturation *in vitro*. Ultimately, the design and conduct of senolytic trials in people with MS require unique cross-disciplinary expertise in neurology, geriatric medicine, and aging biology to integrate aging biomarkers and principles of senolytic treatment with immune response and clinical outcomes in MS (58, 59).

## Conclusion

Evidence implicating cellular senescence—particularly in microglia—as a driver of disease in older individuals with progressive MS has led to the plausible therapeutic strategy of using senolytics to slow disease progression. Preclinical evidence has highlighted the role of senescent microglia in hindering remyelination in the setting of autoimmune neuroinflammation. Senolytics have demonstrated effective clearance of senescent cells; however, their efficacy likely depends on factors such as the specific agent used, as well as the age, sex, and underlying characteristics of the individual. Clinical trials of senolytics in people with MS are currently in development, informed by insights from ongoing and completed studies in other age-related diseases. Targeting cellular senescence is a promising therapeutic approach to address the urgent unmet need for effective treatments in the aging progressive MS population.

## Data Availability

The raw data supporting the conclusions of this article will be made available by the authors, without undue reservation.
